# Long-term case-fatality rate of nontuberculous mycobacterial disease in people living with HIV

**DOI:** 10.1186/s40249-022-00942-8

**Published:** 2022-02-07

**Authors:** Jingjing Hu, Ling Gu, Yueming Shao, Renfang Zhang, Tangkai Qi, Jianjun Sun, Zhenyan Wang, Wei Song, Yang Tang, Jiangrong Wang, Shuibao Xu, Junyang Yang, Yinzhong Shen, Li Liu, Jun Chen, Hongzhou Lu

**Affiliations:** 1 School of Public Health, Bengbu Medical College, Bengbu, 233000 Anhui China; 2 Department of Infectious Diseases and Immunology, Shanghai Public Health Clinical Center, Fudan University, 2901 Caolang Road, Jinshan District, Shanghai, 201508 China; 3 Shenzhen Third People’s Hospital, The Second Affiliated Hospital of Southern University of Science and Technology, Shenzhen, 518000 China

**Keywords:** HIV/AIDS, Nontuberculous mycobacteria, Case-fatality rate, Risk factor

## Abstract

**Background:**

Few data are available regarding the long-term case-fatality rate (CFR) among people living with HIV (PLWH) with nontuberculous mycobacteria (NTM) disease. The aim of this study is to analyze the long-term CFR in patients with NTM disease and to identify risk factors for their death.

**Methods:**

A retrospective cohort study of 379 cases of microbiologically confirmed NTM disease in PLWH was conducted from January 1, 2012, to December 31, 2020, in Shanghai, China. We used Kaplan–Meier survival analysis and the log-rank test to compare the long-term CFR in patients with disseminated NTM (DNTM) and localized NTM disease. Univariate Cox proportional hazards regression analysis and a stepwise Cox proportional hazards regression model were used to estimate the predictors of long-term CFR.

**Results:**

The cohort was followed up for a median of 26 months. The total CFR was 15.7% by one year and increased to 22.6% at 5 years after the diagnosis of NTM disease. The 5-year CFR of PLWH with DNTM was significantly higher than that of PLWH with localized NTM (26.7% vs 19.6% for DNTM and localized NTM disease, respectively). Older age [hazard ratio (HR) = 1.04, 95% confidence interval (*CI*): 1.02–1.06, *P* < 0.001], comorbidity (HR = 2.05, 95% *CI*: 1.21–3.49, *P* < 0.01), DNTM (HR = 2.08, 95% *CI*: 1.17–3.68, *P* < 0.05), and HIV viral load (HR = 1.32, 95% *CI*: 1.12–1.55, *P* < 0.001) were all independent risk factors for long-term CFR. In the subgroup analysis, time to culture positivity was negatively correlated with CFR in patients with DNTM (HR = 0.90, 95% *CI*: 0.82–0.98, *P* < 0.05).

**Conclusions:**

NTM was associated with a high long-term CFR in PLWH. Further approaches to prevent NTM disease in PLWH are urgently needed.

**Graphical Abstract:**

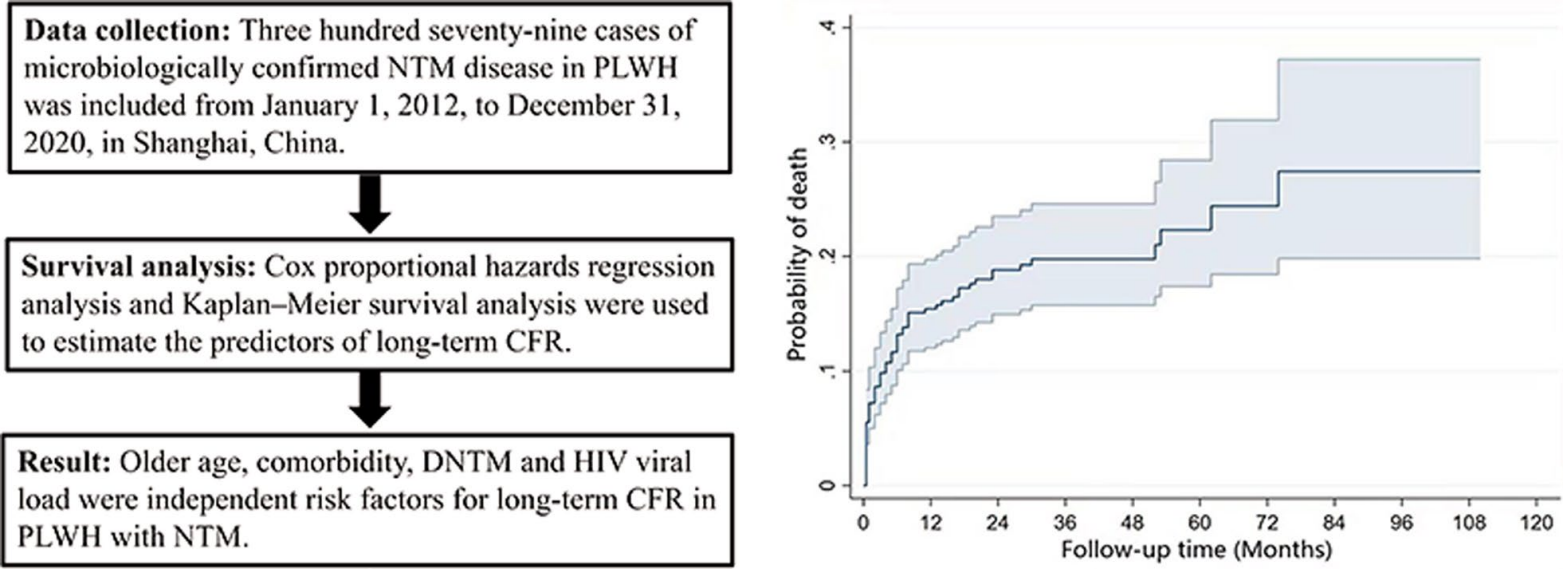

## Background

Nontuberculous mycobacteria (NTM) disease is one of the leading opportunistic infections in people living with HIV (PLWH). Over the past decades, the number of cases of PLWH with NTM has increased with advances in screening techniques, the AIDS epidemic and the increase in the number of immunocompromised patients [1, 2]. Recently, data from the United States showed that the overall prevalence of NTM (among PLWH admitted to the hospital for pneumonia) was 49% (96/196) [3]. In another study, 37 cases of disseminated NTM (DNTM) were identified in 7,349 patients in Oregon, USA, between 2007 and 2012, with a median annual incidence of 110/100,000 HIV person-years and the highest incidence in those with CD4^+^ T cell count < 50 cells/mm^3^ (5,300/100,000 person-years) [4].

In the preantiretroviral therapy era, case fatality rates (CFRs) were high for NTM and even higher for DNTM, with an annual CFR of 71% [5]. Since the antiretroviral therapy (ART) era, AIDS has become a chronic disease, which has led to a significant increase in life expectancy in PLWH, and the incidence of disseminated *Mycobacterium avium* complex (DMAC) has declined significantly from 65.3/1,000 in 1992 to 2.0/1,000 in 2015 [6]. However, despite the availability of effective ART, the CFR for NTM remains high, with a CFR of 69% at 1 year and 27% at 3 years after the diagnosis of DMAC [7].

Modern population-based estimates of the long-term survival of HIV-infected patients with NTM are lacking. Studies have indicated that the long-term survival of PLWH with tuberculosis (TB) is lower than that of non-TB patients [8]. We hypothesized that long-term CFR would also be elevated in PLWH with NTM. Therefore, we conducted this study to analyze the long-term CFR in PLWH with NTM and to identify risk factors for death.

## Methods

### Study subjects

A retrospective analysis was performed on the clinical data of PLWH with NTM in Shanghai Public Health Clinical Center from January 1, 2012, to December 31, 2020. The inclusion criteria were as follows: (1) HIV-1 infection confirmed by Western blotting; (2) patients had at least one specimen with positive mycobacterial culture and negative for MPB64, or the mycobacterial sequencing results were NTM; (3) physicians deemed NTM to be an etiology of the diseases but not colonization. The exclusion criteria were patients younger than 18 years.

### Study design

The following characteristics were recorded: sex, age, behavioral risk factors (such as current or former smoking, alcohol abuse), comorbidities, opportunistic infections, CD4^+^ T cell count, HIV viral load, ART regimen, NTM treatment, symptoms and the time from specimen culture initiation to positive report. Furthermore, we registered the high-resolution computed tomography (CT) scan. All abnormal CT scans were reported and divided into seven categories: (1) lymphadenopathy only; (2) lymphadenopathy with nodules; (3) lymphadenopathy with cavities; (4) lymphadenopathy with cavities and nodules; (5) nodules only; (6) cavities only; (7) and consolidation only. We recorded the laboratory data of all patients, including T-SPOT results, blood cell counts, hemoglobin levels, erythrocyte sedimentation rates, C-reactive protein levels and renal and liver function.

The diagnosis of DNTM was defined as a positive culture for mycobacterium from blood, cerebrospinal fluid, bone marrow, or biopsy of a sterile site or infection involving two or more noncontiguous body sites. Comorbidities were defined based on the Charlson Comorbidity Index [9, 10], which includes 19 major disease categories, including risk factors and potential prognostic factors for PLWH with NTM. Comorbidity data included diabetes, hypertension, cancer, chronic lung disease and other diseases. AIDS-defining opportunistic infections other than NTM infection were also recorded. Baseline CD4^+^ T cell count and HIV viral load were defined using the test results at admission or the closest record at admission. Anti-NTM medication use was defined as drug use for at least 2 weeks.

For the prognosis analysis, survival time was defined as the time from the beginning of definitive diagnosis of NTM to death, loss to follow-up, or the end of follow-up (December 31, 2020). Patients were followed up by telephone after discharge from the hospital. The outcome was all-cause mortality during the follow-up period.

The study was approved by the Ethics Committee of Shanghai Public Health Clinical Center (Ethics approval number: 2020-Y112-01). Informed consent was waived because of the retrospective design of the study.

### Statistical analysis

SPSS statistics 25.0 (IBM, Armonk, NY, USA) and Stata 16.0 (StataCorp LP, College Station, TX, USA) were used for statistical analysis. The Shapiro–Wilk test was used to test whether the data conformed to a normal distribution. Normally distributed data are reported as the means and standard deviation (mean ± SD). Nonnormally distributed data are presented as the medians and interquartile range (IQR). Categorical variables were summarized with frequency counts and presented as a rate (%). The *χ*^2^ test, *t* test and Fisher’s exact test were used to test for statistically significant differences. Kaplan–Meier survival analysis and log-rank test were used to compare long-term CFR in patients with DNTM and localized NTM disease. Univariate Cox proportional hazards regression analysis and stepwise Cox proportional hazards regression models were used to estimate the predictors of long-term CFR. *P* < 0.05 indicated statistical significance. In these analyses, hazard ratios were combined with 95% confidence intervals.

## Results

### Clinical characteristics of the study population

Three hundred seventy-nine patients were included. Of these, 93.7% were male, and the median age was 38.0 (IQR: 30.0–50.0) years. In total, 7.4% of the patients were current or former smokers, and 2.6% consumed alcohol. One hundred thirteen patients (29.8%) had comorbidities, and 136 patients (35.9%) had opportunistic infections. In addition, the median CD4^+^ T cell count was 23.0 (IQR: 6.0–73.8) cells/μl, and the median HIV viral load was 4.84 (IQR: 1.9–5.5) log10 copies/ml. Two hundred ninety-four patients (77.6%) received ART prior to anti-NTM therapy, and the median time from initiation of ART to initiation of anti-NTM therapy was 31.0 (IQR: 4.0–127.0) months (Table 1).

**Table 1:** Baseline characteristics of PLWH with NTM infection

	*n* (%)
General information	
Total number of patients	379
Male sex	355 (93.7%)
Age [years], Median (IQR)	38.0 (30.0–50.0)
Smoking (current or former)	28 (7.4%)
Alcoholism	10 (2.6%)
Comorbidity	113 (29.8%)
Opportunistic infection	136 (35.9%)
Cryptococcosis	35 (25.7%)
Cytomegalovirus	28 (20.6%)
Digestive tract fungal infections	23 (16.9%)
Pneumocystis pneumonia	12 (8.8%)
Talaromyces marneffei	11 (8.1%)
Kaposi’s Sarcoma	8 (5.9%)
Herpes zoster	5 (3.7%)
Mycobacterium tuberculosis	4 (2.9%)
Salmonella infection	3 (2.2%)
Pulmonary aspergillosis	2 (1.5%)
Bacterial pneumonia	2 (1.5%)
Cerebral toxoplasmosis	2 (1.5%)
Progressive multifocal leukoencephalopathy	1 (0.7%)
Clinical manifestations	
Fever	236 (63.4%)
Cough	164 (44.1%)
HIV wasting syndrome	81 (22.8%)
Abdominal pain and diarrhea	68 (18.3%)
Central nervous system symptoms	48 (12.9%)
Rash	43 (11.6%)
HIV-related indicators	
CD4^+^ T cell count [cells/μl], Median (IQR)	23 (6.0–73.8)
HIV viral load [log10 copies/ml], Median (IQR)	4.8 (1.9–5.5)
ART before NTM treatment	294 (77.6%)
ART to anti-NTM time, Median (IQR)	31 (4.0–127.0)
NTM treatment	
Macrolides	280 (73.9%)
Levofloxacin/Moxifloxacin	248 (65.4%)
Ethambutol	317 (83.6%)
Rifampicin/Rifabutin	248 (65.4%)
Linezolid	23 (6.1%)

*PLWH* people living with HIV, *NTM* nontuberculous mycobacteria, *IQR* interquartile range, *HIV* human immunodeficiency virus, *ART* antiretroviral therapy

**Fig. 1:**
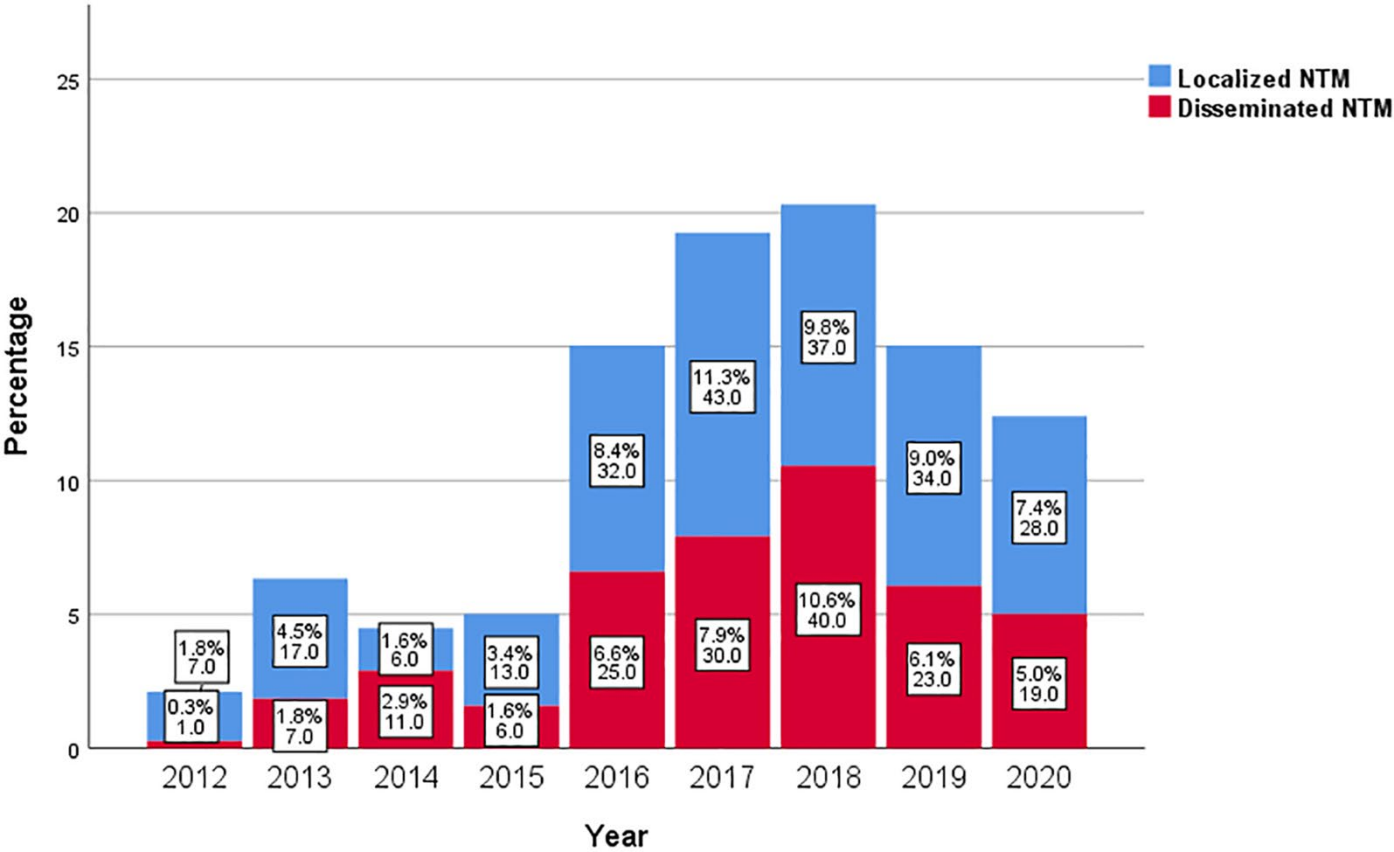
Stacked bar charts of PLWH with disseminated NTM and with localized NTM disease. (The data showed in the charts is percentage and number). *PLWH* people living with HIV, *NTM* nontuberculous mycobacteria

The most frequently reported symptom was fever (63.4%). Cough was reported in 44.1% of all cases. A total of 22.8% reported HIV wasting syndrome, 18.3% reported abdominal pain and/or diarrhea, 12.9% reported central nervous system symptoms such as headache and dizziness, and half of these patients had a combination of cryptococcal meningitis. The remaining 11.6% of patients had skin manifestations, such as rashes (Table 1). For each year from 2013 to 2020, DNTM accounted for almost half of the total number of NTM in PLWH (Fig. 1). Among the first reported positive specimens, sputum accounted for 60.7%; blood accounted for 23.5%; and stool accounted for 6.9%; while the rest were puncture fluid (4.0%), bronchoalveolar lavage or bronchial lavage fluid (1.3%), pleural effusion (0.8%), cerebrospinal fluid (0.8%), bone marrow (0.8%), urine (0.5%), hydroperitoneum (0.3%), abdominal abscess (0.3%) and secretions from ruptured skin (0.3%). The median time to culture positivity was 13.9 (IQR: 9.5–23.5) days.

Three hundred twenty-six (86.0%) patients received CT scans. Of the 326 patients, 148 (45.4%) were described as having lymphadenopathy. The most common morphology was mediastinal lymphadenopathy (128/148, 86.5%) (Table 2).

**Table 2:** Radiology findings of PLWH with NTM infection

		*n* (%)
Lymphadenopathy		148 (45.4%)
Morphology		
	Mediastinal Lymphadenopathy	128 (86.5%)
	Hilar lymphadenopathy	42 (28.4%)
	Retroperitoneal lymph	40 (27.0%)
	Axillary lymph nodes	20 (13.5%)
	Supraclavicular/infraclavicular lymph nodes	8 (5.4%)
	Celiac lymph node	8 (5.4%)
	Pelvic lymph nodes	1 (0.7%)
Categories		
	Lymphadenopathy only	111 (75.0%)
	Lymphadenopathy with nodules	23 (15.5%)
	Lymphadenopathy with cavities	10 (6.8%)
	Lymphadenopathy with cavities and nodules	4 (2.7%)
Nodules		33 (10.1%)
Cavities		14 (4.3%)
Consolidation		3 (0.9%)
Other infectious lesions		107 (32.8%)
No obvious abnormalities		21 (6.4%)

Total of 326 patients received CT scans. *PLWH* people living with HIV, *NTM* nontuberculous mycobacteria, *HIV* human immunodeficiency virus

Treatment for NTM diseases consists of a multidrug regimen and a long course of therapy. Anti-NTM medication for patients included macrolides, levofloxacin/moxifloxacin, ethambutol, rifampicin/rifabutin, and linezolid (Table 1), which lasted for 9–12 months. Almost all of the enrolled patients received ART.

### Survival analysis

**Fig. 2:**
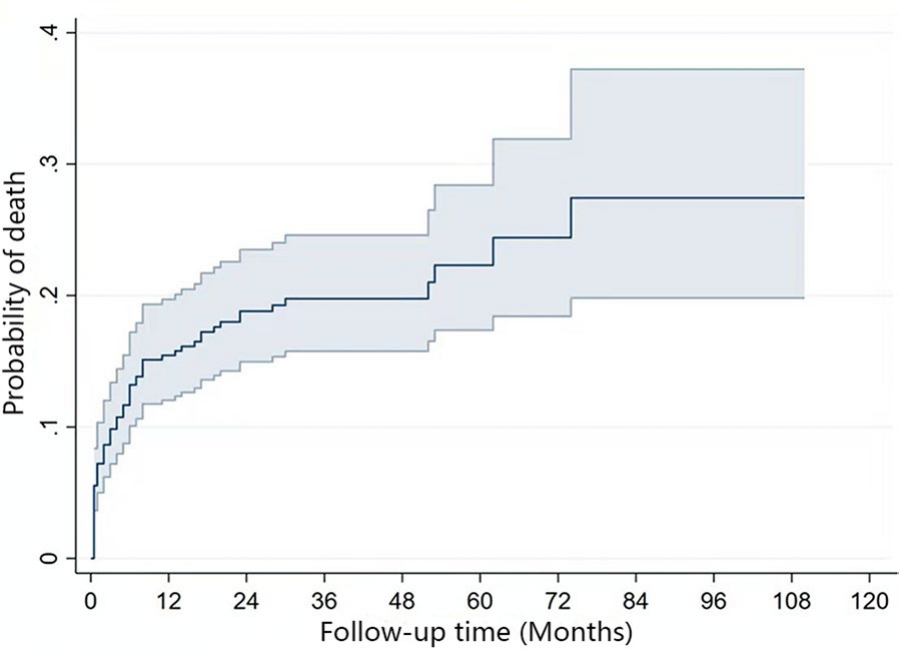
Probability of death (95% confidence interval) among PLWH with NTM. *PLWH* people living with HIV, *NTM* nontuberculous mycobacteria

After a median of 26 months of follow-up, 69 patients (18.2%) died, and 48 (12.7%) were lost to follow-up. In 52.2% of patients, the follow-up period exceeded 2 years. The life table method showed an overall CFR of 15.7% at 1 year, 19.0% at 2 years, 20.0% at 3 years, 22.6% at 5 years, and 27.9% at 7 years. Univariate Cox regression analysis indicated that the following parameters were statistically significant for survival: older age, HIV viral load, ART before NTM treatment, comorbidity, linezolid and DNTM (Table 3). The probability of death in PLWH with NTM increased with time (Fig. 2).

**Table 3:** Hazard ratio in univariate analysis and multivariate analysis

	Univariate analysis			Multivariate analysis		
	Hazard ratio	95% *CI*	*P*	Hazard ratio	95% *CI*	*P*
Age	1.02	1.01–1.04	0.015	1.04	1.02–1.06	0.001
Comorbidity	2.01	1.24–3.23	0.005	2.05	1.21–3.49	0.008
DNTM	1.81	1.12–2.90	0.015	2.08	1.17–3.68	0.012
HIV viral load	1.25	1.07–1.46	0.006	1.32	1.12–1.55	0.001
Linezolid	3.64	1.85–7.15	0.001	4.71	2.25–9.83	0.001
Sex	1.59	0.50–5.04	0.435	–	–	0.697
Smoking	0.86	0.31–2.35	0.764	–	–	0.411
Alcoholism	1.30	0.32–5.29	0.719	–	–	0.592
Opportunistic infection	0.98	0.60–1.61	0.946	–	–	0.411
CD4^+^ T cell count	1.00	1.00–1.00^a^	0.243	–	–	0.795
ART before NTM treatment	0.53	0.32–0.89	0.015	–	–	0.221
Time to culture positivity	0.98	0.96–1.01	0.125	–	–	0.232

Sex, age, smoking, alcoholism, comorbidity, opportunistic infection, CD4^+^ T cell count, HIV viral load, ART before NTM treatment, linezolid, DNTM and time to culture positivity were added to the model using stepwise procedures

–: blank (In the multivariate analysis, SPSS 25.0 did not show 95% *CI* and hazard ratio for variables that were not statistically significant); ^a^: 95% *CI* for hazard ratio of CD4^+^ T cell count: 0.996–1.001

*HIV* human immunodeficiency virus, *NTM* nontuberculous mycobacteria, *DNTM* disseminated nontuberculous mycobacteria, *ART* antiretroviral therapy

**Fig. 3:**
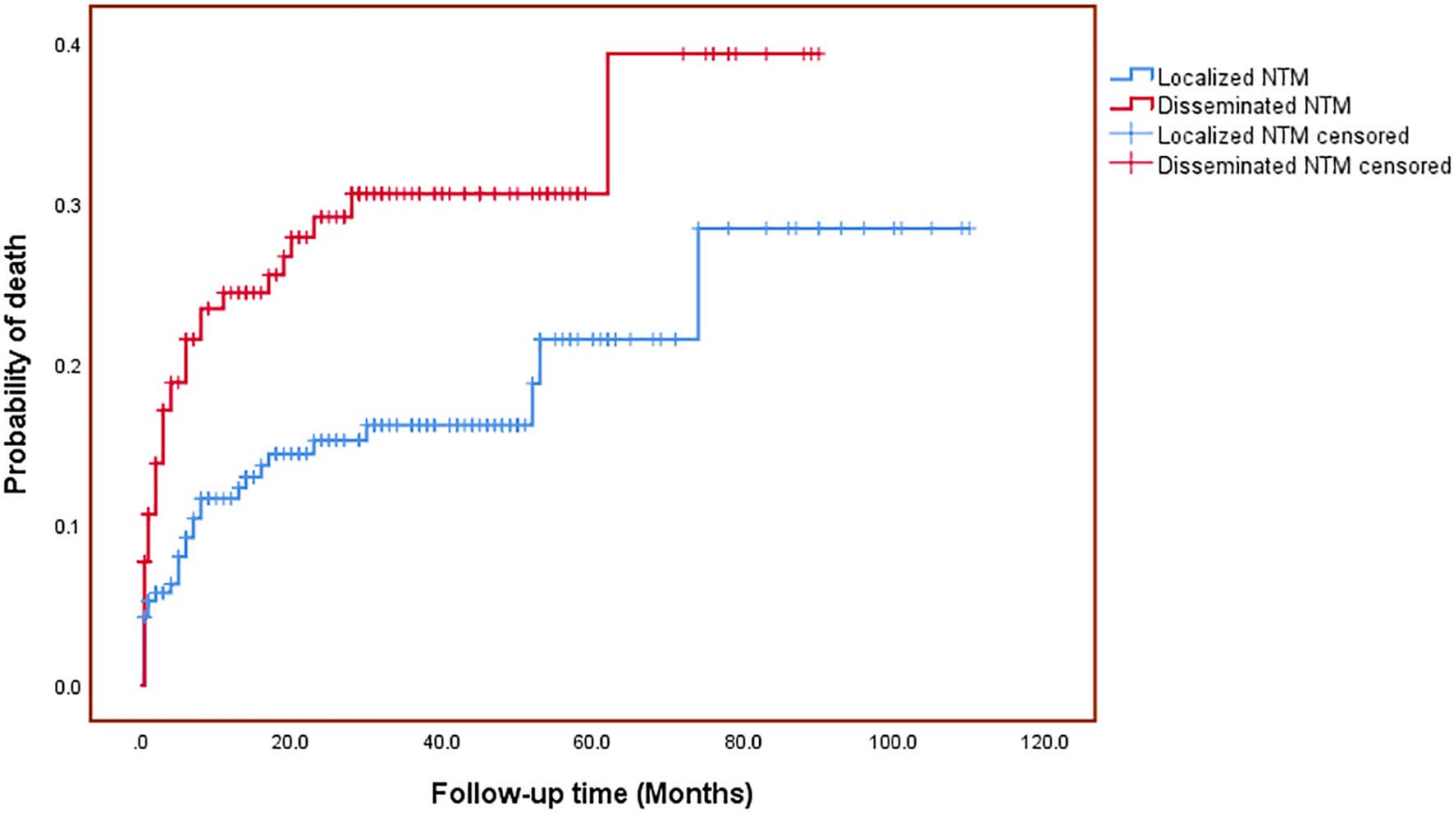
Probability of death among PLWH with disseminated NTM and localized NTM disease. *PLWH* people living with HIV, *NTM* nontuberculous mycobacteria

Considering that sex, smoking, alcoholism, opportunistic infection, CD4^+^ T cell count, and time to culture positivity were also important risk factors, these factors and all parameters that were statistically significant in the univariate analysis were included in a multivariate Cox proportional hazards model. The results showed a hazard ratio of 2.05 [95% confidence interval (*CI*): 1.21–3.49, *P* < 0.01] for patients with comorbidities compared with those without comorbidities. The hazard ratio caused by older age was 1.04 (95% *CI*: 1.02–1.06, *P* < 0.001). High levels of HIV viral load were statistically significant, with a hazard ratio of 1.32 (95% *CI*: 1.12–1.55, *P* < 0.001). DNTM was significantly correlated with poor survival outcomes (HR = 2.08, 95% *CI*: 1.17–3.68, *P* < 0.05) (Table 3). Kaplan–Meier analysis also revealed that the long-term CFR of the DNTM group was significantly higher than that of the localized infection group (Fig. 3). Surprisingly, patients not treated with linezolid had a significantly longer survival time than those treated with linezolid (HR = 4.71, 95%* CI*: 2.25–9.83, *P* < 0.001).

In addition, we performed a stratified analysis by baseline CD4^+^ T cell count. Patients with CD4^+^ T cell counts > 50 cells/μl had CFRs of 7.9%, 12.1%, and 17.9% at 1, 3, and 7 years, respectively. Older age (HR = 1.09, 95% *CI*: 1.04–1.14, *P* < 0.001) and DNTM (HR = 3.52, 95% *CI*: 1.01–12.28, *P* < 0.05) were independent prognostic factors. Patients with CD4^+^ T cell counts ≤ 50 cells/μl had CFRs of 19.9%, 24.4% and 32.7% at 1, 3 and 7 years, respectively. Comorbidities (HR = 2.14, 95% *CI*: 1.19–3.89, *P* < 0.05) and linezolid usage (HR = 2.97, 95% *CI*: 1.34–6.58, *P* < 0.01) were independent prognostic factors. ART before NTM treatment was more beneficial for patients (HR = 0.46, 95% *CI*: 0.25–0.84, *P* < 0.05).

In the subgroup analysis for patients with DNTM, time to culture positivity was negatively correlated with CFR (HR = 0.90, 95% *CI*: 0.84–0.96, *P* < 0.01). The longer the time to a positive culture of the specimen, the lower the number of NTMs, thus favoring survival. Older age (HR = 1.05, 95%* CI:* 1.02–1.08, *P* < 0.01), comorbidity (*HR* = 2.38, 95% *CI*: 1.14–4.96, *P* < 0.05) and linezolid usage (HR = 3.39, 95% *CI*: 1.43–8.02, *P* < 0.01) remained independent risk factors for long-term CFR (Table 4).

**Table 4:** Hazard ratio in multivariate analysis of PLWH with DNTM

	Hazard ratio	95% *CI*	*P*
Age	1.05	1.02–1.08	0.004
Comorbidity	2.38	1.14–4.96	0.021
Linezolid	3.39	1.43–8.02	0.006
Time to culture positivity	0.90	0.84–0.96	0.002

Sex, age, smoking, alcoholism, comorbidity, opportunistic infection, CD4^+^ T cell count, HIV viral load, ART before NTM treatment, linezolid, DNTM and time to culture positivity were added to the model using stepwise procedures

*HIV* human immunodeficiency virus, *ART* antiretroviral therapy, *PLWH* people living with HIV, *DNTM* disseminated nontuberculous mycobacteria

## Discussion

Our study demonstrated that the long-term CFR of PLWH with NTM is high, despite these patients receiving treatment or even recovering. Further analysis revealed that the long-term CFR for disseminated infections was higher than that for those without disseminated infections. Older age, comorbidity, HIV viral load, DNTM and linezolid usage were independent prognostic factors for PLWH with NTM. Although the CD4^+^ T cell count was not significant in the multivariate analysis, we found that patients with CD4^+^ T cell counts ≤ 50 cells/μl had a higher CFR. These patients were more likely to acquire DNTM. For patients with DNTM, the time to culture positivity was negatively correlated with CFR.

Collins et al. studied patients with HIV/AIDS from 1992 to 2015 [7]. Despite effective ART, they found that DMAC infection was associated with a significant increase in CFR in the years following diagnosis. This is similar to the results in our study. Another Japanese study also showed that DNTM was significantly associated with CFR, and the median baseline CD4^+^ T cell count was significantly lower in nonsurvivors than in survivors [11]. However, their sample size of 24 was not very convincing, and further studies are needed.

Data on the impact of NTM on long-term CFR in PLWH are limited, but studies in HIV-negative cohorts have also found high long-term CFR in NTM survivors. Typically, factors such as older age and comorbidities have been reported to be associated with poor prognosis. A systematic review gave an overall estimate of 5-year CFR from NTM pulmonary disease studies. Despite the high heterogeneity of the enrolled studies, the pooled estimate of 5-year all-cause mortality of the 9035 patients was 27% (95% *CI*: 21.3–37.8) [12]. Predictors of CFR that were consistent across studies included male sex, presence of comorbidities and older age of patients [12]. Since the vast majority of PLWH in our cohort were male, there was a gender skew; thus, our data are not quite applicable to the general population.

Several studies have compared long-term CFR among PLWH after completing TB treatment to those without TB [8]. The 5-year CFR for patients who completed TB treatment was 10.2% compared to patients without TB (5.6%) [8]. In our study, the 5-year CFR for NTM was 22.6%, which appears to be more than twice as high as that of TB patients. Chiang et al. also reported that PLWH with DMAC (*n* = 58) had a three times higher 1-year CFR than those with TB (*n* = 98) (48.3% vs 16.3%) [13]. This implies that the long-term prognosis of NTM in PLWH is of critical importance.

Behavioral factors (such as smoking and alcoholism) [14, 15] and various respiratory or nonrespiratory comorbidities [16] increase the risk of acquiring NTM and may partially account for the increased long-term CFR among NTM survivors. Multiple studies in largely HIV-negative populations have documented structural lung defects and impaired pulmonary function after NTM infection, and many studies have found a strong association with increased CFR between a history of NTM and chronic obstructive pulmonary disease and bronchiectasis [17–21]. Recently, Mourad et al. found that the expected survival was reduced by approximately 4 years for a diagnosis of NTM lung disease without comorbidity and by 8.6 years for a diagnosis of NTM lung disease with comorbidity [22]. Among HIV-negative patients with NTM with and without comorbidities, the 5-year CFR after diagnosis was 44.9% and 25.0%, respectively [22].

The high occurrence of disease relapse and increasing drug resistance may lead to an increased CFR. A multicenter study showed that 9.5% of patients with NTM pulmonary infection experienced multiple episodes, with 24.8% of them suffering from relapsing infections caused by the same NTM species [23]. An observational retrospective study from Italy revealed that 35.3% of patients had unsuccessful treatment outcomes, including discontinuation of therapy (13.5%), recurrence (11.2%), reinfection (5.3%), treatment failure (4.1%) and relapse (1.2%) [24]. The treatment of treatment-refractory NTM cases or patients with drug-resistant NTM isolates remains challenging. This may indicate a poor prognosis and high CFR. In clinical practice, patients with NTM infections caused by more resistant species may use linezolid, as their conditions are more severe. This may partially explain why using linezolid was associated with poor outcome in our study.

In addition, NTM may cause persistent inflammation and immune activation, which may increase susceptibility to HIV infection, promote HIV viral replication, and accelerate the progression of HIV disease [25]. As shown in a previous study, 79% (19/24) of PLWH with DNTM had immune reconstitution syndrome, suggesting difficulty in the management of DNTM [11]. Therefore, clinicians should pay high attention to DNTM in PLWH. However, the diagnosis of NTM infection in PLWH is difficult, as the available methods are limited. Notably, nearly half of the patients had abnormal CT imaging in our study, most commonly mediastinal lymphadenopathy. This may give the physician a clue in treating PLWH suspected of NTM infection.

There are some limitations in our study. First, this study was conducted at a single center, and our results may not be generalizable to other regions due to its retrospective nature. Second, we did not use the American Thoracic Society criteria for NTM lung disease. Third, no further species identification was available for most patients. Several studies have found that different mycobacterium species were not significant for CFR analysis [26, 27]. However, a 15-year follow-up study of 1445 patients with NTM pulmonary disease showed that the accurate identification of the species or subspecies of the NTM pathogen is very important in the prognosis [28]. Data from Canada also showed that NTM disease was associated with higher rates of death for all species combined and for most individual species [29]. Therefore, further research on species identification is needed. Furthermore, although the sensitivity of MPB64 is high, a very small percentage of patients may still have a false-negative result [30]. It is possible that a few patients enrolled were due to TB.

## Conclusions

To our knowledge, this is the largest study to date that evaluates the long-term CFR and associated prognostic factors for NTM in PLWH in the modern ART era. Long-term CFR for NTM in PLWH is high. Older age, comorbidity and DNTM are independent prognostic factors for NTM. These findings highlight the critical importance of PLWH with NTM and suggest that PLWH with a history of NTM may require closer long-term follow-up.

## Data Availability

The datasets used and/or analyzed during the current study are available from the corresponding author on reasonable request.

## Abbreviations

CFR: Case-fatality rate
HIV: Human immunodeficiency virus
PLWH: People living with HIV
NTM: Nontuberculous mycobacteria
DNTM: Disseminated nontuberculous mycobacteria
MAC: *Mycobacterium avium* Complex
DMAC: Disseminated *Mycobacterium avium* complex
ART: Antiretroviral therapy
TB: Tuberculosis
CT: Computed tomography
SD: Standard deviation
IQR: Interquartile range
